# Early-life microbiota-immune homeostasis

**DOI:** 10.3389/fimmu.2023.1266876

**Published:** 2023-10-23

**Authors:** Hayley M. Reynolds, Matthew L. Bettini

**Affiliations:** Department of Microbiology and Immunology, University of Utah, Salt Lake, UT, United States

**Keywords:** gut microbiota, dysbiosis, early-life, probiotics, prebiotics, fecal microbiota transplant, immune homeostasis

## Abstract

As the prevalence of allergy and autoimmune disease in industrialized societies continues to rise, improving our understanding of the mechanistic roles behind microbiota-immune homeostasis has become critical for informing therapeutic interventions in cases of dysbiosis. Of particular importance, are alterations to intestinal microbiota occurring within the critical neonatal window, during which the immune system is highly vulnerable to environmental exposures. This review will highlight recent literature concerning mechanisms of early-life microbiota-immune homeostasis as well as discuss the potential for therapeutics in restoring dysbiosis in early life.

## Introduction

The neonatal intestinal microbiome, composed of bacteria, fungi, viruses, protozoa and archaea, is in constant flux from initial microbial seeding to approximately three years of age, when the microbiota stabilizes and more closely resembles adult-type microbial composition (1, 2). During this period of flux, many factors can influence colonization.

**Figure 1:**
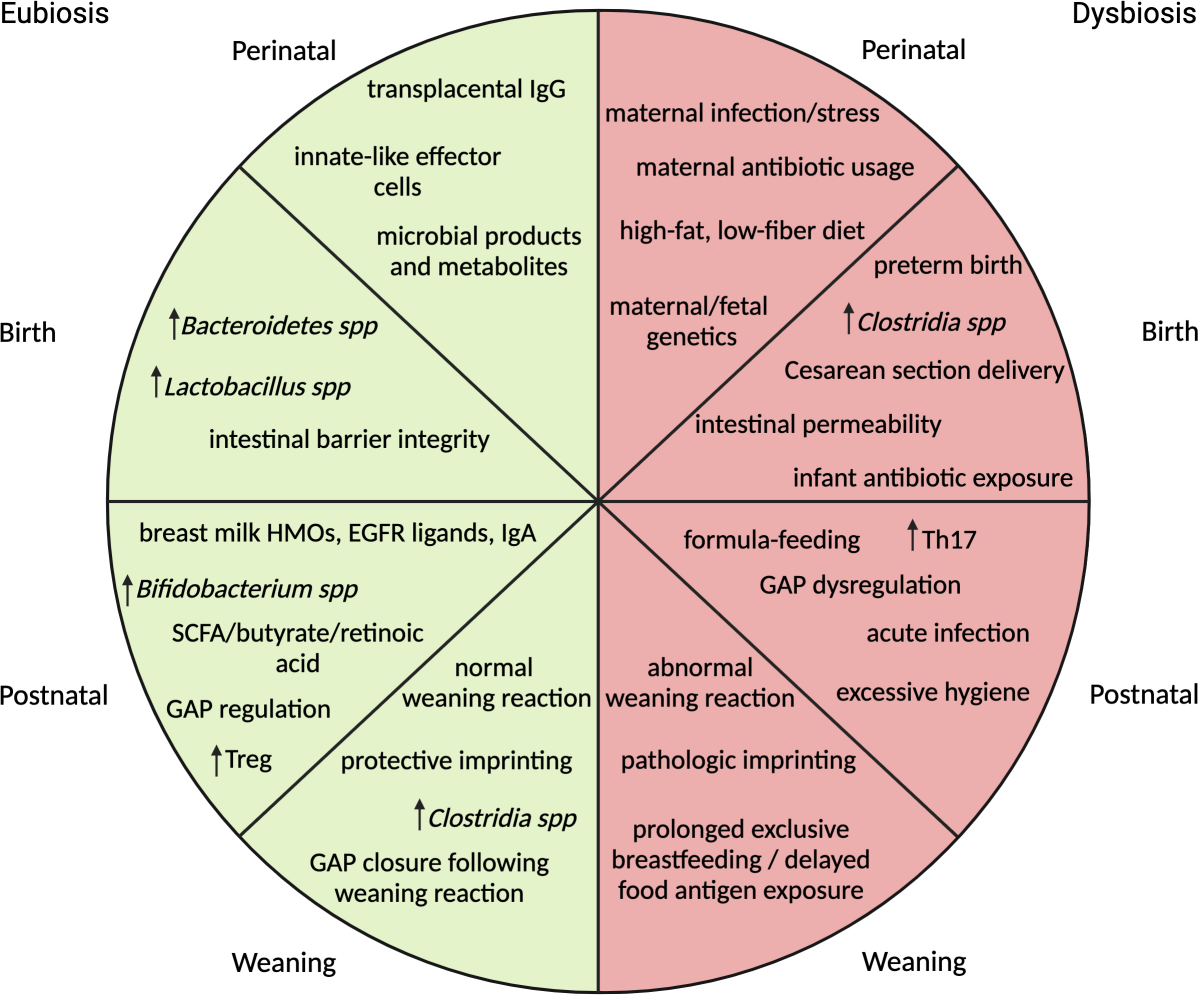
Summary of events impacting microbial composition and immune maturation across different stages of early-life development correlated with association for eubiosis or dysbiosis. In the perinatal stage, maternal environmental influences and immunity are the primary drivers of fetal immune development. At birth, gestational age, mode of delivery, environmental stressors and exposure to antibiotics impact initial microbial colonizers and influence intestinal barrier permeability. In the postnatal period, composition of diet and environment determine makeup of microbial communities, regulate timing of GAP opening and influence immune cell differentiation. At weaning, exposure to solid foods brings about microbial expansion followed by a vigorous immune response or weaning reaction. If the reaction is absent or delayed, pathologic immune imprinting may occur. (HMO-human milk oligosaccharides, EGFR-epidermal growth factor receptor, SCFA-short chain fatty acids, GAP- goblet cell associated antigen passages).

The timeline of initial microbial seeding is a matter of debate. The sterile womb theory, adopted over a century ago, suggests that seeding does not occur until birth (3). In recent years, challengers to this theory provide evidence of microbial products in the placenta, amniotic fluid, umbilical cord blood and fetal membranes however discord remains due to the inability to culture viable bacteria from these sources (4–7). Regardless of the exact time of colonization, many factors are capable of influencing microbiome composition during development (**Figure 1**). Prior to birth, maternal stress, diet and infection have all been shown to associate with alterations in the microbiome (1, 8–13). During birth, the microbiome is influenced by factors like gestational age, mode of delivery and delivery environment (2, 14–18). Following birth, antimicrobial exposure, infant stressors, consumption of breast milk or formula, timing of solid food introduction and the childrearing environment may continue to shape early-life microbiota development (2, 19–23). Additionally, the genetic makeup of the neonate is implicated in gut microbiome composition by altering the host immune response or nutrient metabolism (24, 25).

As microbes compete for space and nutrients, the developing immune system is faced with the challenge of defending the newborn from pathogens while ensuring tolerance toward beneficial microbes. Transplacental IgG antibodies offer passive immunity as well as immune priming prior to birth (26). Following birth, breast milk products play a key role for the microbial transition from birth to weaning. Breast milk contains human milk oligosaccharides (HMOs) which provide selective advantage to microbes such as *Bifidobacterium infantis* (27, 28). Additionally, breast milk-derived IgA promotes colonization of select microbes by facilitating their adherence to the intestinal epithelium (29). Goblet cell-associated antigen passages or GAPs allow for translocation of microbes and antigens from the intestinal lumen to lamina propria antigen presenting cells (APCs) (30). GAP formation is inhibited by epidermal growth factor receptor (EGFR) ligands which are found in high abundance in breast milk after parturition but diminish over time, allowing for a controlled exposure to luminal antigen sensing once the neonate’s immune system is further developed (31, 32). During weaning, solid food introduction results in a large increase in bacterial numbers and mice undergo a weaning reaction or vigorous immune response resulting in tolerogenic imprinting in the context of eubiosis or pathological imprinting in the context of dysbiosis (33). Tolerogenic imprinting requires the generation of RORγt^+^ T regulatory cells which are induced by the activation of the STAT3 transcription factor following bacterial-derived short chain fatty acid (SCFA) and retinoic acid exposure in combination with bacterial antigen presentation by MHC class II (MHCII) on dendritic cells (34). Compared to RORγt^+^ Tregs that develop in adulthood, those generated early in life are long-lived and are capable of providing continued tolerance to microbial and food antigens (35).

Factors disrupting the carefully timed progression of microbial colonization and immune development can result in increased risk for atopy and autoimmune disease. Rising rates of allergy and autoimmune disease associated with industrialization have prompted theories that seek to link environmental influences with immune impact. The two prevailing hypotheses are the “hygiene hypothesis” proposed by David Strachan in 1989 and the “old friends” hypothesis by Graham Rook in 2003 (36–38). The hygiene hypothesis posits that an absence of sufficient microbial exposure early in life can lead to increased susceptibility to disease, while the old friends hypothesis suggests that exposure to evolutionarily conserved microbial species during development is required for training of the immune system to react appropriately to pathogens.

In recent decades, many studies aim to delve into more mechanistic understandings of microbial-immune interactions. However, the complexity of these interactions, inter-facility variation in gut microbiome, limited resolution of 16S rRNA sequencing and the simplified microbiota of laboratory mice have posed a challenge to these efforts. Moving forward, multi-omic approaches and the introduction of additional mouse models such as the ‘wilding mice’ or humanized mice may help to enhance the reproducibility and translational potential of research findings in this field.

This review will focus on current literature investigating the impact of early-life microbial exposures on the developing immune system and mechanisms of microbial restoration during early-life dysbiosis.

## Mechanisms of microbial-immune homeostasis

### Protective immunity

In addition to protection provided by maternal factors, the neonatal immune system is quickly developing to help eliminate acute pathogenic threats. The first two waves of hematopoiesis from the yolk sac and fetal liver bring about a slew of innate effectors consisting of macrophages, mast cells, Natural Killer (NK) cells, innate B-1 cells, dendritic cells, innate lymphoid cells (ILCs), γδ T-cells, and mucosal-associated invariant T (MAIT) cells (39). Many of these cells undergo imprinting in early life that is dependent on the microbial environment.

Unconventional T-cell subsets which include MR1-restricted MAIT cells, CD1d-restricted NKT cells and CD1a/c/d-restricted γδ T-cells have been shown to be regulated by early-life microbial exposure and metabolites (40). By using the PLZF transcription factor to identify innate-like lymphocytes in mice, it was demonstrated that early-life antibiotic usage can decrease the frequency of PLZF-expressing cells in the thymus, a phenomenon that persists into adulthood (41). Antibiotics given in adult life did not impact thymic PLZF^+^ populations suggesting that the time frame for microbial influence is specific to early life (41). Likewise, a separate group showed that colonization of neonatal germ-free mice with riboflavin-synthesizing microbes induced MAIT cell development at barrier sites, but colonization of adult mice was not sufficient to generate these cells (42). The degree to which dysfunctional development of unconventional T-cells may influence disease is an area of ongoing research. A study in germ-free mice demonstrated accumulation of invariant NK T (iNKT) cells in colonic lamina propria and lung that resulted in increased morbidity for inducible models of IBD and allergic asthma (43). Conventional microbiota colonization of neonatal mice but not adult mice was sufficient to prevent iNKT accumulation and pathology (43) A different study found neonatal mice treated with broad-spectrum antibiotics developed an increased susceptibility for imiquimod-induced psoriasis mediated by γδ+ T-cells (44). Interestingly, mice treated with the same antibiotic regimen as adults demonstrated ameliorated psoriasis in comparison to the control group (44).

In addition to unconventional T-cells, unconventional B-cells also play a role in neonatal defense. B-1 cells are innate-like B-cells that develop in the neonatal period and express a highly cross-reactive BCR repertoire which binds both self and microbial antigens. In mice, exposure to bacterial antigens during a specific neonatal period is important for establishing clonal diversity in the GlcNAc-reactive B-1 repertoire (45). Whether this repertoire contributes to homeostatic mucosal IgA responses that can impact microbial colonization in adulthood is yet to be determined (46). In human neonates, polyreactive innate-like B-cells were shown to generate antibodies that are capable of binding commensal bacteria and may facilitate intestinal colonization (47).

Similar to B-1 cells, early-life CD8^+^ T-cells derived from fetal liver have been recognized as possessing distinct transcriptional profiles that favor low TCR diversity and increased rates of homeostatic proliferation (48, 49). This comes at the expense of memory formation but allows for an enhanced innate-like ability to respond broadly to inflammatory signaling. Historically, these cells were thought to be immature, but newer literature suggests these cells are intentionally poised to meet the needs of the neonate and contribute to rapid effector response in adulthood (49, 50). The microbiota also contributes to signaling that allows for protective immunity by CD8^+^ T-cells. In the postnatal period, the dominant conventional dendritic cells are of the cDC1 subtype (51). Upon exposure to microbiota at birth, monocytes and macrophages release myeloid-derived tumor necrosis factor (TNF) (52). This signal modifies fatty acid metabolism in neonatal pre-cDC1 and promotes maturation and increased IL-12p40 production that is important for promoting protective CD8^+^ T-cell responses (52). Maternal antibiotic treatment delays cDC1 differentiation and maintains cDC1s in a tolerogenic IL-10 producing state resulting in increased neonatal susceptibility to pathogens such as *Listeria monocytogenes* (52).

Altogether, early-life microbiota exposure is important for the maturation and function of cells that provide protective immunity to the developing neonate and disruption in microbial exposure may leave infants vulnerable to infection.

### Tolerance

While strong effector responses are critical in protecting the vulnerable infant during development, a hyper-reactive immune response is equally dangerous and thus tolerogenic adaptive immunity quickly follows. Toward the end of gestational development, progenitors from the fetal liver and bone marrow seed the thymus and generate conventional CD4^+^ T-cells (39). In early life, the CD4^+^ T-cell response is skewed toward a Th2 and FOXP3-expressing Treg phenotype (53). Though the contribution of thymic-derived (tTregs) and peripherally-induced (pTregs) is debated due to lack of differentiating phenotypic surface markers, it is clear that both populations are important for suppressing effector T-cell responses to microbiota. Therefore, understanding the mechanisms by which these cells develop may provide important clues as to how tolerance is generated in the neonatal period.

In the intestine, RORγt^+^ pTregs have been shown to mediate tolerance toward commensal microbes and are selectively decreased in germ-free and antibiotic-treated mice (34, 54). Recently discovered, the APCs responsible for RORγt^+^ pTreg differentiation during the weaning reaction were found to display features of both medullary thymic epithelial cells and dendritic cells. These “thetis cells” expressing transcription factors RORγt and AIRE, were found to be enriched between 1-3 weeks of age and then quickly declined. Temporal ablation of MHCII on RORγt expressing APCs in early life but not adulthood resulted in a decline in pTregs (55). pTregs generated during this critical window imprint a durable microbiota-specific T-cell response which persists into adulthood, again suggesting a tightly regulated developmental window with long-lasting implications for the adult immune response (55). In addition to traditional intestinal APCs, a separate study indicated the importance of the intestinal epithelium in promoting pTreg induction while dampening commensal-specific Th17 responses (56). It was shown that microbiota-sensitive epithelial histone deacetylase (HDAC3) is capable of regulating MHCII expression on inter-epithelial cells (IECs). Loss of either epithelial HDAC3 or MHCII on IECs resulted in a significant reduction of commensal-specific Tregs and increase in commensal-specific Th17 cells (56).

Thymic-derived tTregs are also important for neonatal tolerance against microbes colonizing the skin and may also play a role in intestinal microbial tolerance (57, 58). By performing high-throughput TCR sequencing on mice with limited TCR repertoires, it was shown that tTregs constitute the majority of Tregs in intestinal organs (58). How intestinal microbial antigens may influence selection of T-cells in the thymus is still an outstanding question in the field, but recent work suggests that in early life, CX3CR1^+^ APCs can migrate from the intestine to the thymus to influence thymic microbiota-specific T-cell development (59). This area of research warrants further study because it is still unclear how signaling events downstream of interactions between developing T-cells and their cognate peptide-MHC ligands determine the threshold of TCR reactivity and T-cell responses toward commensal microbes later in life.

Anti-inflammatory signaling molecules are also important for maintaining tolerance in early life and can be influenced by microbial colonization. Transforming growth factor-β (TGF-β) is a pleiotropic cytokine which regulates IgA production and promotes the suppressor functions of both pTregs and tTregs (60, 61). TGF-β1 levels are reduced in the colon of germ-free mice as compared to *Clostridia-*colonized mice suggesting a role for microbiota in regulating its expression (62). Recent literature has identified the SCFA, butyrate, as the primary metabolite which acts to increase TGF-β production in intestinal epithelial cells via HDAC class 1 inhibition (63). Another example of microbial contribution to tolerance is the ability of the *Bacteroides* genus to enhance pTreg function and IL-10 expression by signaling through toll-like receptor 2 (TLR2) via polysaccharide A (PSA) (64).

While this section primarily focuses on Tregs during development, other cell types such as myeloid-derived suppressor cells (MDSCs) and B regulatory cells can also contribute to immune tolerance in the neonatal period and future studies may demonstrate how their development is impacted by early-life microbiota.

Given that this phase of development is centered around tolerance, vaccines administered at birth generally have reduced efficacy. Additionally, immunization at birth with diphtheria, pertussis and tetanus (DTaP) was associated with a significant reduction in antibody response to subsequent boosters compared with a routine immunization schedule suggesting that early neonatal vaccination might impair the response later in life (65). This being the case, the majority of vaccinations are administered after the neonatal period around 2 months of age, but with the substantial burden of respiratory syncytial virus (RSV)-associated hospitalizations, there is increasing demand for earlier and more effective intervention. Solutions being investigated include utilizing adjuvants that better engage the infant immune system and encouraging maternal vaccination to provide passive protection (66, 67). Another consideration for vaccination efficacy, is the tolerogenic response to environmental microbial exposures that may preclude vaccination efforts (68, 69). For example, the lowest efficacy rates of the Bacille Calmette-Guérin (BCG) vaccine against tuberculosis is seen in equatorial regions where exposure to environmental mycobacteria is common. A study investigating this phenomenon in mice found that they could circumvent this immunosuppressive response by changing the route of vaccine administration from oral to pulmonary (70).

Both protective and tolerogenic immune responses are important for healthy neonatal development but their imbalance may increase infection susceptibility or increase propensity to develop allergy and autoimmune disease. Alterations to microbiota exposure or composition in the neonatal period can have long-lasting consequences that impact immunity in adult life. Importantly, these disruptions cannot always be resolved once the critical window of opportunity closes. Multiple factors may result in deviation from the carefully constructed developmental pathway, so what options are there to restore early-life microbiota in the face of dysbiosis?

## Therapeutics

This section will discuss attempts to restore normal microbiota in common scenarios which may alter microbial composition during early life such as antibiotic treatment, preterm birth, cesarean section delivery (CSD) and formula-feeding.

Fecal microbiota transplantation (FMT) is a procedure during which fecal matter is transferred from a healthy donor to a recipient in order to restore microbial homeostasis in the gut. The technique’s origins can be traced back to 4^th^ century China where patients with food poisoning or severe diarrhea were given a “yellow soup” composed of human feces. Today, FMT has been approved by the FDA for use in scenarios of recurrent *Clostridioides difficile* infection and ongoing research may highlight additional clinical applications.

Currently, FMT is being explored for use in infants that are born via CSD. Cesarean-born infants have been shown to differ in microbial composition with increases in skin-associated populations such as *Staphylococcus* as well as increases in *Clostridia spp* (71). CSD is also associated with increased risk for allergy, atopy and asthma suggesting that the composition of early seeding is important for immune development (72). Efforts have been made to correct for aberrant microbial exposures by exposing infants to maternal vaginal fluid topically and orally but have had little success integrating maternal strains into the intestinal fetal microbiome (73). A proof-of-concept study found that maternal FMT in Cesarean-born infants restores normal intestinal microbial flora, particularly replenishing *Bacteroidales* spp. abundance (74). Though promising, the study also observed that a significant portion of mothers’ stool tested positive for pathogenic microbes such as group B *Streptococcus.* As a result, these samples were excluded from the study and highlight the need for careful screening to ensure the safety of FMT.

Prebiotics and probiotics represent alternatives to FMT that may be more easily regulated and standardized. Due to the importance of human milk oligosaccharides in promoting expansion of beneficial early-life microbes, a randomized, controlled, double-blind trial was performed to study the benefits of supplementing standard cow’s milk-based formula with HMOs, using a separate non-randomized human milk-fed group as reference (75). The report follows infants from enrollment (baseline) until 6 months of age, taking fecal samples for analysis at baseline, 3 and 6 months of age. Using shotgun metagenomics, the study found increases in relative abundance of *Bifidobacterium* spp. including *B. infantis* at 3 and 6 months in feces of groups receiving the formula with HMOs compared to those receiving the control formula. Overall, HMO-supplemented formula shifted microbial composition closer to that of infants who received breast milk suggesting that HMO supplementation has the potential to normalize microbiota and immune maturation in formula-fed infants (75).

An alternative to prebiotic supplementation is to administer beneficial microbes directly (probiotics). Probiotic administration in neonates is most popular in the context of preterm birth with the most common bacterial species reintroduced being *Lactobacillus* and *Bifidobacterium*. Preterm birth is associated with a variety of complications that can increase the risk of neonatal death. One these complications, necrotizing enterocolitis (NEC) is a gastrointestinal disease characterized by ischemic necrosis of the intestinal mucosa and inflammation which can lead to bowel perforation and sepsis in severe cases. NEC is associated with increase abundance of pathobionts within the phylum *Proteobacteria* (family *Enterobacteriaceae*) (76). Studies show that probiotic administration in preterm infants is capable of reducing the relative abundance of these species but may not affect longitudinal gut microbiota composition (77, 78). Despite this encouraging data, the efficacy of probiotic usage in preventing NEC remains inconclusive and requires further study (79).

Ongoing clinical studies hope to shed more light on the efficacy of these microbial restoration strategies to help inform best clinical practice when faced with early-life dysbiosis (80–89).

## Discussion

While many studies focus on reversing the progression of allergy and autoimmune disease later in life, prevention and early correction of dysbiosis during the neonatal window is important for minimizing the public health burden of disease. Despite major advances in this field of research, many outstanding questions remain. As the utilization of neonatal microbial restoration interventions becomes more popular, future studies should seek to better understand their impact on the developing immune system and subsequent potential to reduce the risk of developing dysbiosis-related disease. Finally, although the timeline for the window of opportunity in mice is more or less defined, how that translates to the specific timeline of human neonatal development remains unclear given that the human neonate has a more mature immune landscape at birth compared with neonatal mice (53). Defining these time points more precisely may help to inform clinical recommendations on when best to introduce complementary foods and administer vaccines during development and may also help to determine the timeframe during which methods of correcting dysbiosis will be most effective.

## Author contributions

HR: Conceptualization, Funding acquisition, Writing – original draft, Writing – review & editing. MB: Conceptualization, Funding acquisition, Supervision, Writing – review & editing.
